# Activation of Bilateral Secondary Somatosensory Cortex With Right Hand Touch Stimulation: A Meta-Analysis of Functional Neuroimaging Studies

**DOI:** 10.3389/fneur.2018.01129

**Published:** 2019-01-10

**Authors:** Gemma Lamp, Peter Goodin, Susan Palmer, Essie Low, Ayla Barutchu, Leeanne M. Carey

**Affiliations:** ^1^Neurorehabilitation and Recovery, Florey Institute of Neuroscience and Mental Health, Melbourne Brain Centre, Heidelberg, VIC, Australia; ^2^Occupational Therapy, School of Allied Health, La Trobe University, Bundoora, VIC, Australia; ^3^Department of Neurology, Sunshine Hospital, Western Health, Melbourne, VIC, Australia; ^4^Department of Psychology, Royal Adelaide Hospital, Adelaide, SA, Australia; ^5^Balliol College, University of Oxford, Oxford, United Kingdom

**Keywords:** ALE “activation likelihood estimation”, meta-analysis, brain activation, sensation, hand, touch, secondary somatosensory cortex

## Abstract

**Background:** Brain regions involved in processing somatosensory information have been well documented through lesion, post-mortem, animal, and more recently, structural and functional neuroimaging studies. Functional neuroimaging studies characterize brain activation related to somatosensory processing; yet a meta-analysis synthesis of these findings is currently lacking and in-depth knowledge of the regions involved in somatosensory-related tasks may also be confounded by motor influences.

**Objectives:** Our Activation Likelihood Estimate (ALE) meta-analysis sought to quantify brain regions that are involved in the tactile processing of the right (RH) and left hands (LH) separately, with the exclusion of motor related activity.

**Methods:** The majority of studies (*n* = 41) measured activation associated with RH tactile stimulation. RH activation studies were grouped into those which conducted whole-brain analyses (*n* = 29) and those which examined specific regions of interest (ROI; *n* = 12). Few studies examined LH activation, though all were whole-brain studies (*N* = 7).

**Results:** Meta-analysis of brain activation associated with RH tactile stimulation (whole-brain studies) revealed large clusters of activation in the left primary somatosensory cortex (S1) and bilaterally in the secondary somatosensory cortex (S2; including parietal operculum) and supramarginal gyrus (SMG), as well as the left anterior cingulate. Comparison between findings from RH whole-brain and ROI studies revealed activation as expected, but restricted primarily to S1 and S2 regions. Further, preliminary analyses of LH stimulation studies only, revealed two small clusters within the right S1 and S2 regions, likely limited due to the small number of studies. Contrast analyses revealed the one area of overlap for RH and LH, was right secondary somatosensory region.

**Conclusions:** Findings from the whole-brain meta-analysis of right hand tactile stimulation emphasize the importance of taking into consideration bilateral activation, particularly in secondary somatosensory cortex. Further, the right parietal operculum/S2 region was commonly activated for right and left hand tactile stimulation, suggesting a lateralized pattern of somatosensory activation in right secondary somatosensory region. Implications for further research and for possible differences in right and left hemispheric stroke lesions are discussed.

## Introduction

Somatosensory function is crucial for daily life, guiding our interactions with the world around us through the detection, discrimination and recognition of body sensations (1). Somatosensation is important not only for perception, but also for goal-directed action (2, 3). For example, somatosensation contributes to the fundamental pinch grip-lift-and hold task (4) and is important in dexterous movement of the hand (5). Following stroke, reduced functional arm use is contributed to by motor *and* somatosensory deficits. Somatosensory impairment has a negative impact on grasp and manipulation of objects (6) and is associated with reduced arm use (7). Further, somatosensory brain regions have been implicated in motor recovery (8). It has been suggested that somatosensory processing for the guidance of action can be dissociated from the processing that leads to perception (2). Here we focus on brain regions involved in somatosensation, specifically tactile stimulation of the hand, without motor confounds.

The neuroanatomy of somatosensory processing is well established through a large body of lesion, post-mortem, animal and structural neuroimaging studies (9–12). Reproducible functional activation in the contralateral primary somatosensory cortex (S1) has been demonstrated in healthy controls when asked to perceive a touch stimulus to their fingertips (13). Technological advances in recent years have even allowed mapping of individual fingers to corresponding areas of S1 (14) and the temporal acuity of anticipation of a tactile stimulus originating in the ipsilateral S1 (15).

Different patterns of activation and lateralization emerge when examining somatosensory processing in the secondary somatosensory cortex (S2). Median nerve stimulation has been shown to activate bilateral S2 regions, including parietal operculum, regardless of the hand being stimulated, but only the contralateral S1 (16). This has also been seen in other stimulation studies. Lee et al. (17) recently examined the differential neural activations associated with vibrotactile, pressure and temperature stimulation of right palm, showing common activation in the contralateral S1 and bilateral S2/insula regardless of stimulation type. Bilateral S2 region activation has also been seen with vibrotactile stimulation irrespective of other cognitive demands (18). It has been suggested that serial somatosensory processing occurs from contralateral S1 to contralateral S2 in response to electrical stimulation, but when stimulation becomes more intense or painful there is an increase in hemispheric integration (19). A meta-analysis of studies examining the functional role of S2 in somatosensory processing divided the area into OP1 (parietal operculum 1), OP2, OP3, and OP4 (10). While OP1 is reported to represent the human homolog of macaque area S2 and was generally more responsive to pure somatosensory (tactile) stimuli (10), overall the areas were all implicated in different somatosensory processes (20). A thorough review of the functional role of S2, from the bi-laterality of activation with unilateral stimulation, to the mapping of the hand area spread of OP1-OP4, has been provided by Eickhoff et al. (10).

When examining the literature it becomes clear that the functional activation of somatosensory processing in the brain is still a developing area. There are various stimulation techniques to investigate reflexive neural activity, for example vibrotactile stimulation (18, 21) as opposed to MNS median nerve stimulation (16, 22, 23), that can yield different results. Somatosensory stimuli are applied to various body parts, including the face, upper limb, and lower limb (10, 24), but may not be performed on each hand separately (25, 26). Finally, studies have often been confounded by motor contributions to the task, e.g., involving movement intention and/or execution (27–29).

Our aim was to characterize and synthesize the somatosensory brain activation network during touch sensation, with potential influence of motor contributions eliminated. We employed the ALE meta-analytic technique to provide a statistically-based likelihood estimation of the brain regions that are consistently activated during tactile stimulation of the hands. Firstly, studies were limited to those that involved only tactile stimulation of the right (RH) or left hand (LH) separately in order to allow interpretation of networks that account for hemispheric dominance. Following this, studies which incorporated any motor movements during the stimulation task were excluded, to address confounding motor influence during somatosensory task performance. Lastly, to characterize neural correlates specific to touch sensation, studies involving other somatosensory modalities, such as pain or proprioception, were excluded.

## Methods

### Identification of Studies for Meta-Analysis

**Figure 1:**
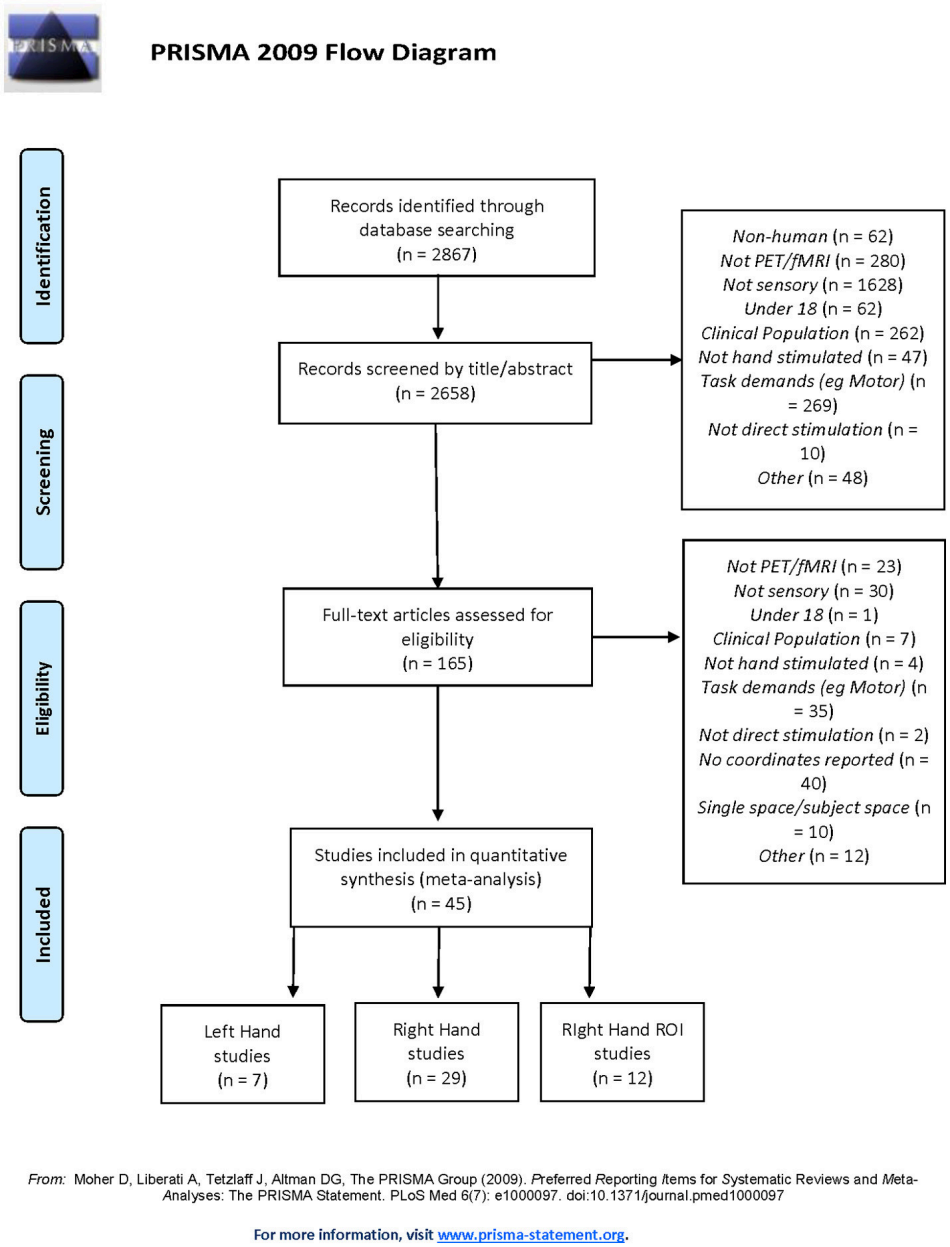
PRISMA Flowchart of selection criteria for including and excluding studies.

The meta-analysis of neuroimaging studies was conducted according to the PRISMA statement and recorded using the suggested checklist (30). A thorough literature search was conducted using Web of Science database (conducted December 12, 2017) and the following search terms: (fMRI OR MRI OR PET OR “functional magnetic resonance imaging” OR “positron emission tomography” OR neuroimaging OR “brain imaging” OR “neural activation”) AND (somatosen^*^ OR sens^*^ OR tactile) AND (hand OR “upper limb” OR finger) AND (health^*^ OR control). These papers were then crosschecked with papers identified in the Sleuth functional database (31–33). The Sleuth database was searched for “somesthesis perception” in the behavioral domain and for “activation only” studies. These were reviewed using the strict inclusion criteria (see Figure 1).

### Activation Likelihood Estimation Meta-Analysis

The meta-analysis was performed using Activation Likelihood Estimation (ALE) on the activation voxel coordinates reported by the selected study (34–36). Analyses were conducted using GingerALE (version 2.3.6) (37) software (downloaded from http://brainmap.org/ale), with coordinates in Montreal Neurological Institute (MNI) space (38, 39). Coordinates reported in Talairach space (40) were converted to MNI space using the “icbm2tal transform” (41, 42). To minimize within-experiment and within-group effects we utilized the modified algorithm described in Turkeltaub, Eickhoff (36) and, thus, were able to include multiple contrasts from within the one study. The calculated ALE map had a cluster forming threshold of *p* < 0.001 with 1000 permutations, corrected for multiple comparisons using the Family Wise Error (FWE) *p* < 0.05 (20, 37, 43, 44). Contrast and conjunction analyses were calculated to compare activation associated with task type, first by creating an image of two tasks pooled together (e.g., RH and LH) with an uncorrected threshold of *p* < 0.01 at 10,000 permutations, and then subtracting each original task analysis from the pooled image in an iterative process (45, 46). Contrast analyses permitted identification of regions of difference between groups while conjunction analyses quantify regions of overlap. To maximize accurate localization and interpretation, images created in GingerALE were also imported into the SPM Anatomy Toolbox (47–49) to permit localization of the ALE images with 3-dimensional probabilistic cytoarchitectonic mapping (50, 51). This regional cytoarchitectonic classification of ALE maps complements the GingerALE localization that uses peak MNI co-ordinates.

## Results

A total of *n* = 45 studies were determined to be suitable for inclusion (see Table 1). Of the 45 studies, 29 were used to perform the RH whole-brain meta-analysis, seven were used for the LH whole-brain meta-analysis (three studies involved stimulation of both LH and RH independently), and 12 studies examined RH stimulation in a ROI analysis.

**Table 1:** Studies included in the meta-analysis (*n* = 45) and reported participant and task information, separated by task category.

**References**	***N***	**Age M (SD); min-max**	**Sex M:F**	**Handedness**	**Stimulus type**	**Stimulus Location**	**Attended**	**Response required**	**fMRI/PET**
**Right Hand stimulation Whole-Brain (*N =* 29)**									
Borstad et al. (52)	10	39–82	5:5	9RH, 1LH	Brush stroke	Index finger	Y	N	fMRI
Bjornsdotter et al. (53)	22	19–35	13:9	NR	Brush stroke	Palm	Y	N	fMRI
Brodoehl et al. (54)	34	21–71	17:17	RH	Compressed air	Fingers 1-3	Y	N	fMRI
Brodoehl et al. (55)	10	23.1 (1.54)	0:10	RH	Compressed ait	Fingers 1-5	Y	N	fMRI
Brodoehl et al. (56)	32	21–71	15:17	RH	Compressed air	Fingers 1-5	Y	N	fMRI
Burton et al. (57)	11	19–25	5:6	RH	Textured surface	Digits 2-3	Y	Y (after scan)	fMRI
Carey et al. (13)^*^	5	52–76	3:2	RH	Texture grids	Fingertips	Y	Y (after scan)	PET
Chung et al. (58)	21	24.19 (2.71)	NR	RH	Band pressure	Index finger	Y	N	fMRI
Chung et al. (59)	21	24.19 (2.17)	NR	RH	Band pressure	Index finger	Y	N	fMRI
Gelnar et al. (27)	9	18-NR	NR	RH	Vibration	Fingers 2-5	Y	N	fMRI
Godde et al. (60)	10	18–30	8:2	RH	Vibration	Fingers	Y	N	fMRI
Hagen et al. (61)^*^	18	37 (12)	11:7	RH	Von Frey	Index finger	Y	N	PET
Hlushchuk and Hari, (62)	10	23–33	7:3	NR	Compressed air	Index, middle, ring fingers	Y	N	fMRI
Kavounoudias et al. (63)	10	31.4 (10.7)	2:8	RH	Textured surface	Whole hand	Y	N	fMRI
Kitada et al. (64)	5	23–25	5:0	RH	Pressure	First 2 fingers	Y	Y	fMRI
Kitada et al. (65)	14	23–26	12:2	RH	Tactile grids	2 Fingers	Y	N	fMRI
Kwon et al. (66)	10	25.20 (2.49); 22–29	5:5	RH	Rubber brush	Dorsum	Y	N	fMRI
Lee et al. (17)	10	27.8 (4.1); 23–34	8:2	NR	Vibratory brush	Palm of right hand	y	N	fMRI
Malinen et al. (67)	10	20–32	6:4	RH	Vibration	Fingers 2-3	NR	NR	fMRI
McGlone et al. (68)	10	18–26	0:10	RH	Brush stroke	Palm	NR	NR	PET
Nebel et al. (69)	12	28.7 (7.6)	0:12	NA	Vibration	Hand	NR	N	fMRI
Ozcan et al. (70)^*^	12	22–35	8:4	11RH, 1LH	Compressed air	Fingertips	N	N	fMRI
Planetta and Servos, (71)	10	25 (1)	3:7	RH	Pressure	Fingertips	NR	NR	fMRI
Rolls et al. (72)	9	28 (NR)	5:4	RH	Textured surface	Hand	NR	NR	fMRI
Ruben et al. (73)	8	21–31	6:2	NR	Electrical stimulation	Digit 2 and 5	NR	NR	fMRI
Schurmann et al. (74)	13	22–39	9:4	RH	Vibration; Compressed air	Hand; Fingers	Y	N	fMRI
Summers et al. (75)	6	20–33	6:0	RH	Vibration	Digit 2	Y	N	fMRI
Yoo et al. (76)	13	21–38	8:5	RH	Von Frey brush	Index finger	Y	N	fMRI
Young et al. (77)	10	21–32	6:4	RH	Textured surface	Hand	Y	N	fMRI
**Right Hand stimulation Region of Interest (ROI) studies (*N =* 12)**									
Blankenburg et al. (78)	8	25–39	7:1	RH	Electrical stimulation	Third finger and palm	NR	N	fMRI
Blatow et al. (79)	12	25–59	5:7	RH	Vibration	Digits 1 and 2	Y	Y	fMRI
Blatow et al. (80)	16	21–51	8:8	RH	Vibration	Digits 1 and 2	NR	NR	fMRI
Burton et al. (81)	12	28.3 (12.8)	8:4	RH	Vibration	Index finger	Y	Y	fMRI
Deuchert et al. (82)	8	23–26	4:4	RH	Von Frey monofilaments	Thenar eminence	Y	Y	fMRI
Dresel et al. (83)	6	24–39	2:4	5RH, 1LH	Electrical stimulation	2 and 5th finger	N	N	fMRI
Eickhoff et al. (10)	14	25.6 (3.4)	7:7	RH	Brush stroke	Fingers	Y	Y	fMRI
Hlushchuk and Hari, (62)	6	20–30	2:4	RH	Compressed air	Palm	NR	NR	fMRI
Huang and Sereno, (84)	9	23–33	6:3	NR	Compressed air	Digits 2,3,4	Y	N	fMRI
Kobayashi et al. (85)	10	18–22	0:10	RH	Textured surface	Palm	Y	N	fMRI
Martuzzi et al. (86)	10	20–35	10:0	RH	Stroke	Finger tips	Y	N	fMRI
Nelson and Chen, (87)	12	25–66	4:8	RH	Vibration	Fingertip	Y	N	fMRI
**Left Hand stimulation Whole-Brain (*N* =7)**									
Ackerley et al. (88)	12	18–35	12:0	NR	Brush stroke	Palm	Y	N	fMRI
Carey et al. (13)^*^	5	33–80	2:3	RH	Texture grids	Fingertips	Y	Y (after scan)	PET
Case et al. (89)	26	24.8 (7); 19–43	11:15	RH	Brush stroke	Palm and back of hand	Y	N	fMRI
Hagen et al. (61)^*^	12	39 (13)	6:6	11RH, 1LH	Von Frey	Index finger	Y	N	PET
Maldjian et al. (90)	5	28–40	4:1	RH	Vibration	Each finger pad	NR	NR	fMRI
Ozcan et al. (70)^*^	12	22–35	8:4	11RH, 1LH	Compressed air	Fingertips	N	N	fMRI
Wacker et al. (91)	13	22–35	9:4	12RH, 1LH	Vibration	Index finger	Y	N	fMRI

* *Studies contributing data to both RH and LH stimulation Whole-Brain analyses*.

As can be seen in Table 1, for the 29 RH whole-brain studies, a total of *n* = 375 participants were included (*n* = 173 males, however *n* = 3 studies did not report sex) aged 18–76 years. The RH ROI studies included *n* = 123 participants (*n* = 63 males) aged 18–66 years. The seven LH studies included *n* = 85 participants (*n* = 52 males) aged 18–80 years. The most common form of stimulation was vibration (*n* = 12 studies), followed by compressed air (*n* = 8), textures (*n* = 7), brush stroke (*n* = 7), Von Frey filaments (*n* = 4), and pressure (*n* = 4).

**Figure 2:**
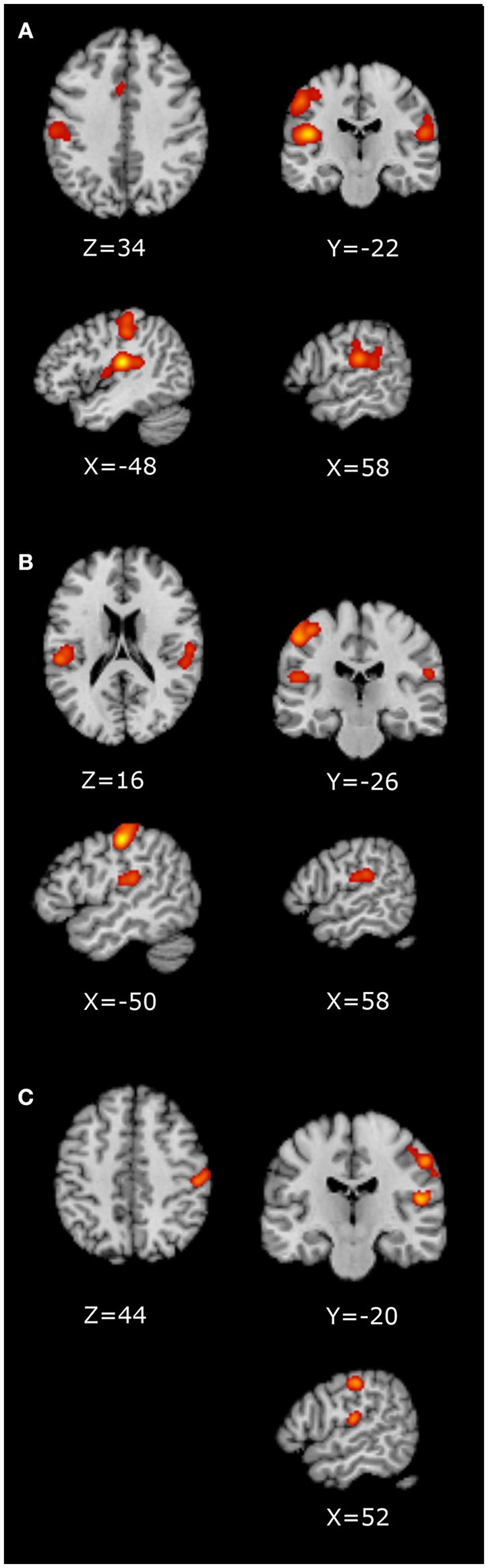
ALE Images displayed in neurological convention. **(A)** RH whole-brain ALE; **(B)** RH ROI ALE; **(C)** LH whole-brain ALE.

The RH whole-brain studies, RH ROI studies and the LH whole-brain studies were analyzed separately, as presented in Table 2 and Figure 2. For the RH whole-brain stimulation studies, the contralateral (left) primary and secondary somatosensory areas were significant, with a large cluster containing the parietal operculum (92), somatosensory (93), and motor (94) cortices. The ipsilateral (right) secondary somatosensory cortex, S2, was also significant, largely comprising the parietal operculum (92) and inferior parietal cortex (95, 96), in addition to a small cluster in the anterior cingulate. The RH ROI studies revealed visually smaller contralateral (left) clusters in the primary and secondary somatosensory regions, with a smaller ipsilateral (right) cluster within S2. The contralateral (left) clusters were separated into a large superior cluster containing the primary somatosensory (93) and motor (94) cortices, and a smaller inferior cluster containing primarily the parietal operculum (92). The ipsilateral (right) cluster contained similar areas to RH whole brain, namely the parietal operculum (92) and inferior parietal cortex (95, 96). With the small number of LH stimulation studies, only two clusters were significant in the contralateral (right) primary (containing somatosensory (93) and motor (94) cortices) and secondary somatosensory regions [primarily parietal operculum (92)] and primary auditory cortex (97) (Table 2).

**Table 2:** Anatomical location, summary statistics and MNI co-ordinates of ALE identified areas for RH whole-brain, RH ROI and LH whole-brain studies (Extrema ALE value, FWE cluster corrected *p* < 0.05, uncorrected *p* < 0.001).

**SPM Anatomy Toolbox region location**	**MNI GingerALE peak location**	**Extrema value**	**Size**	***x***	***y***	***z***
**RH WHOLE-BRAIN STUDIES (30 CONTRASTS, 334 FOCI)**						
Left parietal operculum (OP) Area OP3 (VS), area OP4 (PV), and area OP1 (S2)	Left primary somatosensory area (S1); Insula (BA 13)	0.061623	17,784	−48	−20	20
*Left Area 1, Area 3b, and Area 4a*	*Left S1; postcentral gyrus (BA 2)*	*0.035296*		*−54*	*−20*	*48*
*Left Area 3b, Area 1, and Area 4a*	*Left S1; postcentral gyrus (BA2)*	*0.028759*		*−44*	*−26*	*58*
*Not assigned in probability maps*	*Left Insula (BA 13); claustrum*	*0.021434*		*−38*	*−12*	*4*
*Left Area OP4 (PV)*	*Left primary motor area (M1); insula (BA 13)*	*0.016304*		*−44*	*−8*	*10*
*Not assigned in probability maps*	*Left par opercularis (BA 44); insula (BA 13)*	*0.015817*		*−40*	*4*	*10*
Right area OP1 (S2), area OP4 (PV), and area TE 1.0	Right supra marginal gyrus (SMG, BA 40); insula (BA 13)	0.039009	6,032	56	−22	20
*Right area PFcm (inferior parietal lobe, IPL), and Area OP1 (S2)*	*Right superior temporal area (BA 22); insula (BA 13)*	*0.021011*		*56*	*−34*	*18*
*Right Area PFcm (IPL) and Area PF (IPL)*	*Right IPL, SMG (BA 40)*	*0.017283*		*56*	*−38*	*28*
*Right area PFop (IPL), area PFt (IPL), and area 3b*	*Right S1; postcentral gyrus (BA 2)*	*0.015576*		*60*	*−20*	*32*
Left area 33	Left cingulate gyrus (BA 24, 32)	0.022505	896	−4	14	36
**RH ROI STUDIES (12 CONTRASTS, 93 FOCI)**						
Left area 1, area 4a	Left S1; postcentral gyrus (BA 2)	0.025885	6520	−50	−18	52
*Left area 4a and area 3b*	*Left M1; postcentral gyrus (BA 3)*	*0.017133*		*−40*	*−28*	*60*
*Left area 4a and 3b*	*Left M1; postcentral gyrus (BA 3)*	*0.015225*		*−42*	*−22*	*58*
Left area OP1 (S2), area TE 1.0, and area PFop (IPL)	Left postcentral gyrus, SMG, BA 40)	0.014924	2,296	−54	−26	20
*Left area OP4 (PV), area OP3 (VS), and area OP1 (S2)*	*Left S1; insula (BA 13)*	*0.012088*		*−50*	*−20*	*20*
*Area OP3 (VS) and Area OP4 (PV)*	*Left M1; Insula (BA 13)*	*0.007544*		*−42*	*−12*	*16*
Right Area OP1 (S2), Area PFcm (IPL), and Area PFop (IPL)	Right IPL, SMG (BA 4)	0.012395	1,840	54	−26	24
*Right area OP1 (S2) and area OP4 (PV)*	*Right SMG (BA 40); insula (BA 13)*	*0.011046*		*58*	*−18*	*20*
*Right area OP4 (PV)*	*Right S1, postcentral gyrus (BA 43)*	*0.007434*		*60*	*−8*	*14*
**LH WHOLE-BRAIN (7 CONTRASTS, 53 FOCI)**						
Right Area 1, Area 3b, and Area 4p	Right primary somatosensory area (S1); postcentral gyrus (BA2)	0.013074	3,176	54	−20	44
*Right Area 3b, Area 4p, and Area 4a*	*Right S1; IPL (BA40), postcentral gyrus*	*0.010481*		*40*	*−34*	*60*
*Right Area 1 and Area 3b*	*Right S1; postcentral gyrus (BA 3)*	*0.009132*		*44*	*−24*	*64*
*Right Area 1, Area 3b, and Area PFt (IPL)*	*Right S1; postcentral gyrus (BA 3)*	*0.008071*		*62*	*−18*	*36*
*Right Area 4p, Area 3b, and Area 3*	*Right S1; IPL (BA 40)*	*0.007689*		*36*	*−34*	*52*
*Not assigned in probability maps*	*Right M1; precentral gyrus (BA 4)*	*0.007664*		*44*	*−12*	*60*
Right area OP4 (PV), area OP1 and area TE 1.0	Right supramarginal gyrus (SMG: BA 40); Insula (BA 13)	0.016235	1,392	52	−16	16

*MNI, Montreal Neurological Institute; ALE, Activation Likelihood Estimation; RH, Right Hand; ROI, Region of Interest; LH, Left Hand; FEW, Family Wise Error; SPM Anatomy Toolbox location based on 3 dimensional probabilistic cytoarchitectonic maps (47–51); MNI GingerALE peak location based on anatomical location of peak MNI co-ordinate from the GingerALE software; (OP), Parietal Operculum; OP3 (VS), Ventral Somatosensory; OP4 (PV), Parietal Ventral; OP1 (S2), Second Somatosensory; Area TE 1.0, Central Primary Auditory Cortex (PAC); PFcm, IP within Parietal Operculum; IPL, Inferior Parietal Lobe; PF, Caudal inferior parietal cortex (IPC); PFop, Rostro-ventral IPC; PFt, Dorsal IPC; BA, Brodmann Area. Locations in italics refer to areas within the larger clusters (i.e. sub clusters identified)*.

**Figure 3:**
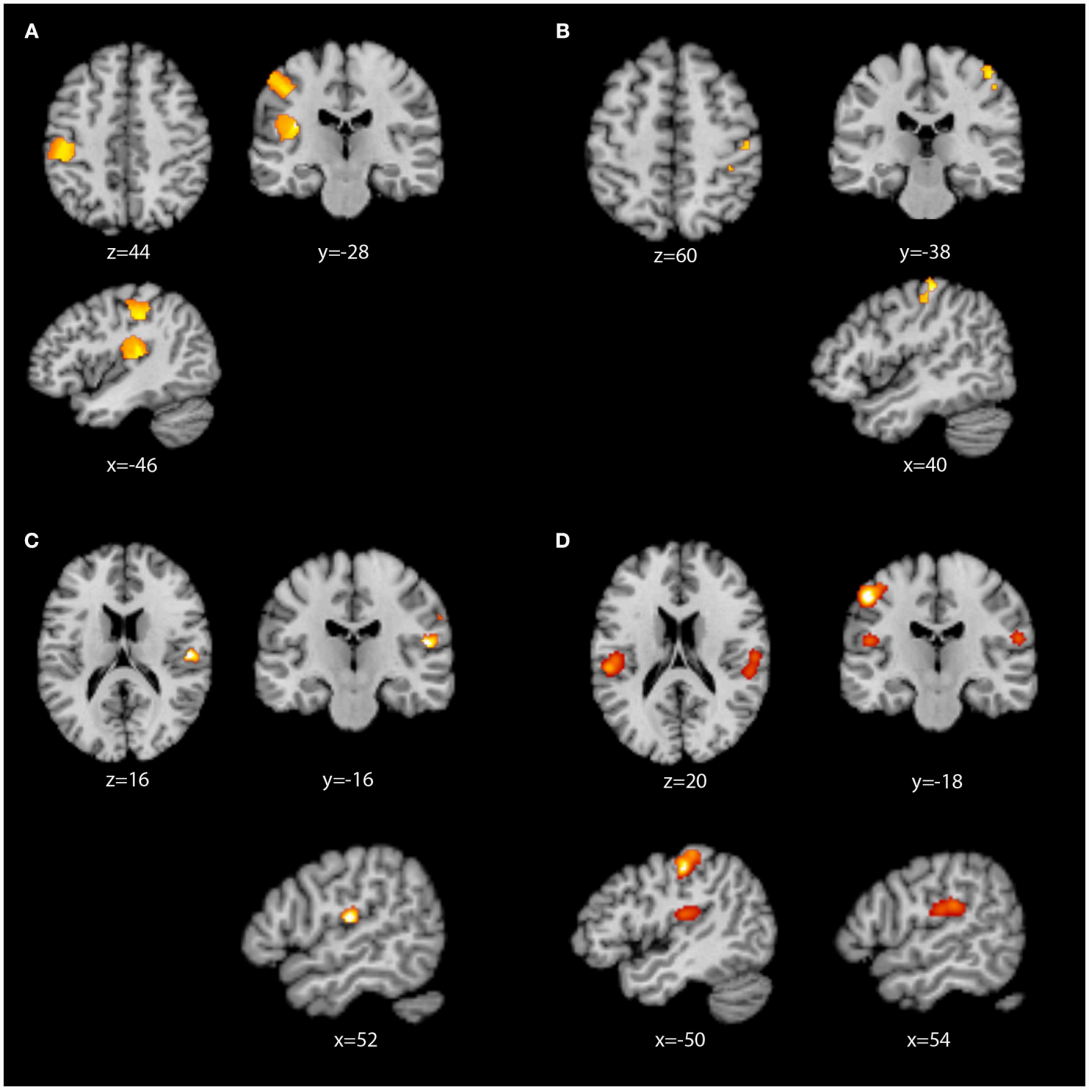
ALE images displayed in neurological convention. **(A)** RH whole-brain activation greater than LH whole-brain activation; **(B)** LH whole-brain activation greater than RH whole-brain activation; **(C)** RH whole-brain conjoined with LH whole brain activation; **(D)** RH whole-brain conjoined with RH ROI activation.

Contrast analyses were then performed, as presented in Table 3 and Figure 3. When contrasted with the LH whole-brain studies, RH whole-brain studies revealed two clusters in the contralateral (left) primary and secondary somatosensory areas. The largest cluster contained primary somatosensory (93, 98) and motor (94) cortices, while the smaller cluster contained primarily the auditory cortex (97), insula (99), and parietal operculum (92). When contrasted with RH whole-brain studies, the LH whole-brain studies activated three small clusters in S1 quite similar to those found in the standalone LH whole-brain analysis, all containing primary somatosensory areas (93, 98). In our analysis of conjoined areas (i.e. areas of overlap) for RH and LH whole-brain studies, only one significant cluster was present, in the right secondary somatosensory region, primarily parietal operculum areas OP1, OP3 and OP4 (92). There were no significant differences in the contrast analysis between RH whole-brain studies and RH ROI studies. However, when the two groups were conjoined, significant common regions of activation were identified, with clusters revealed in the left primary (93) and secondary somatosensory areas (92), and the right secondary somatosensory area (92), including OP1.

**Table 3:** Anatomical location, summary statistics and MNI coordinates of ALE identified areas for contrast analyses: RH Whole-Brain greater than LH Whole-Brain, LH Whole-Brain greater than RH Whole-Brain, RH Whole-Brain conjoined with LH Whole-Brain, and RH Whole-Brain conjoined with RH ROI studies (*p* < 0.01, 10,000 *p*-value permutations, 100 mm cluster threshold).

**SPM Anatomy Toolbox region location**	**MNI GingerALE peak location**	**Extrema value**	**Size**	**x**	**y**	**z**
**RH WHOLE-BRAIN GREATER THAN LH WHOLE-BRAIN STUDIES**						
Left area 3b, area 2 and area 4p	Left Inferior parietal lobe (IPL), supramarginal gyrus (SMG: BA 40)	3.719017	4,168	−45	−28	44
*Left area 4p, area 4a, and area 3a*	*Left S1: postcentral gyrus (BA 2)*	*3.540084*		*−49*	*−25*	*50*
*Left area 4a and area 1*	*Left S1; postcentral gyrus (BA 2)*	*3.352795*		*−52*	*−19*	*53*
Left area TE 1.0, area lg2, and area TE 1.1	Left transverse temporal gyrus (BA 41)	3.890592	3,488	−39	−22	17
*Left area TE 1.0, area TE 1.1, and area OP1 (S2)*	*Left transverse temporal gyrus (BA 41)*	*3.719017*		*−45*	*−26*	*16*
*Left area lg2, area TE 1.2, and area TE 1.0*	*Left S1; insula (BA 13)*	*3.352795*		*−44*	*−18*	*12*
*Not assigned in probability maps*	*Left S1; postcentral gyrus (BA 2)*	*3.011454*		*−44*	*−20*	*28*
**LH WHOLE-BRAIN GREATER THAN RH WHOLE-BRAIN STUDIES**						
Right area 3b and area 2	Right S1: IPL (BA 40)	2.597153	296	40	−38	60
*Right area 3b, area 4p, and area 2*	*Right S1; IPL (BA 40)*	*2.582808*		*36*	*−36*	*54*
*Not assigned in probability maps*	*Right S1; postcentral gyrus (BA 40)*	*2.483769*		*40*	*−30*	*58*
Right area 1 and area 3b	Right postcentral gyrus (BA 3)	2.894304	288	45	−26	58
Right area 1 and area 3b	Right postcentral gyrus (BA 3)	2.911238	280	48	−22	56
*Right area 1 and area 3b*	*Right postcentral gyrus (BA 3)*	*2.847963*		*52*	*−20*	*52*
*Right area 3b and area 4a*	*Right postcentral gyrus (BA 2)*	*2.575829*		*48*	*−18*	*54*
*Not assigned in probability maps*	*Right postcentral gyrus (BA 40)*	*2.536396*		*47*	*−21*	*50*
**RH WHOLE-BRAIN STUDIES CONJOINED WITH LH WHOLE-BRAIN STUDIES**						
Right area OP4 (PV), area OP1 (S2), and area OP3 (V5)	Right SMG BA 40); insula (BA 13)	0.016235	688	52	−16	16
**RH WHOLE-BRAIN CONJOINED WITH RH ROI STUDIES**						
Left area 3b, area 4a, and area 1	Left S1; postcentral gyrus (BA 2)	0.025433	4,400	−50	−18	50
*Not assigned in probability maps*	*Left M1; postcentral gyrus (BA 3)*	*0.017133*		*−40*	*−28*	*60*
*Not assigned in probability maps*	*Left M1; postcentral gyrus (BA 3)*	*0.015225*		*−42*	*−22*	*58*
Left area OP1 (S2), area TE 1.0, and area OP4 (PV)	Left postcentral gyrus, SMG (BA 40)	0.014924	2,144	−54	−26	20
*Not assigned in probability maps*	*Left S1; insula (BA 13)*	*0.012088*		*−50*	*−20*	*20*
*Not assigned in probability map*s	*Left M1; insula (BA 13)*	*0.007544*		*−42*	*−12*	*16*
Right area OP1 (S2) and area OP4 (PV)	Right IPL, SMG (BA 40)	0.012395	1,632	54	−26	24
*Not assigned in probability maps*	*Right insula (BA 13), SMG (BA 40)*	*0.011046*		*58*	*−18*	*20*

*MNI, Montreal Neurological Institute; ALE, Activation Likelihood Estimation; RH, Right Hand; ROI, Region of Interest; LH, Left Hand; SPM Anatomy Toolbox location based on 3 dimensional probabilistic cytoarchitectonic maps (47–51); MNI GingerALE peak location based on anatomical location of peak MNI co-ordinate from the GingerALE software; Area TE 1.0 - Central Primary Auditory Cortex (PAC); Ig2 - Granular Insula area 2; TE 1.1 - Medial PAC; TE 1.2 - Lateral PAC; OP4 (PV) - Parietal Ventral; OP1 (S2) – Second Somatosensory; OP3 (VS), Ventral Somatosensory; BA, Brodmann Area. Locations in italics refer to areas within the larger clusters (i.e. sub clusters identified)*.

## Discussion

In two important ways our ALE meta-analysis allowed us to examine the brain regions consistently activated during tactile stimulation of the hands in order to characterize functional somatosensory regions and networks, without the influence of motor function. Firstly, the meta-analysis allowed us to characterize and compare areas involved in right hand and left hand tactile stimulation studies separately. Secondly, it revealed the similarities and differences between functional activation studies that focus on specific brain regions (RH ROI studies) and what is actually occurring throughout the brain (RH whole-brain studies). Unfortunately very few studies (*n* = 7) examined LH stimulation separate to the RH and without the influence of motor activity, making a statistical comparison between the hands difficult and exploratory.

For the RH whole-brain stimulation studies (*n* = 29) not only did we find two large clusters in the contralateral (left) primary (93) and secondary [specifically within parietal operculum areas OP1, OP3 and OP4 (92)] somatosensory cortices as expected, but activation was also revealed in the ipsilateral (right) secondary somatosensory region involving OP1 and OP4 (92) in addition to the anterior cingulate. Bilateral activation of secondary somatosensory S2 region, involving parietal operculum (92) to unilateral stimulation of the right hand is consistent with previous reports (100). From the few LH studies included, two small but significant clusters were revealed in the contralateral (right) S1 and S2. While each hand had significantly greater activation in the contralateral S1 and S2 in comparison to the other hand, the only significant area of overlap was in the right S2, specifically OP1, OP3 and OP4 (92). Lateralized differences have been reported for different sensory modalities, with right hemisphere being more spatially oriented toward the dorsal perceptual/sensory systems (101). Overlap in right S2 is consistent with hemispheric asymmetry involving right-hemisphere-based bilateral representation of the body (101), right-sided asymmetry for tactile processing (102) and robust bilateral responses to unilateral stimulation in S2 (100). Due to the difference in numbers of studies included for each hand, this comparison is considered exploratory and highlights the need for more studies to examine LH tactile stimulation separately. Nevertheless, it is an interesting trend and could have significant implications for better understanding somatosensory function and dysfunction.

Activation in the contralateral S1, when using a tactile stimulus on the hand, is quite consistent with previous research (13, 14). The pattern of activation shown in the RH whole-brain studies is consistent with research showing contralateral S1 activation only, *and* studies that have shown bilateral activation in S2 regardless of the hand being stimulated (16, 17, 103). It is surprising that bilateral S2 activation was not seen for the meta-analysis of LH studies also. However, this may have been attributable to the low number of studies stimulating the LH alone.

The role of S2 both contralateral and ipsilateral to the hand being stimulated is particularly interesting and may have important implications. The secondary somatosensory cortex of nonhuman primates is located on the parietal operculum, and the anatomical cytoarchitectonic maps of OP 1-4 of the human parietal operculum correlate with the functionally defined human somatosensory cortex (92), with OP 1 constituting the putative human homologue of area S2 (92). Further, OP1 is closely connected with the parietal networks for higher order somatosensory processing, while OP 4 is more closely integrated with areas responsible for basic sensorimotor processing and action control (104). Bilateral secondary somatosensory cortex, in particular, has demonstrated a role in complex integrative processes of stimulus elaboration and attention following stimulation of right hand (105). Tame, Braun (103) have demonstrated bilateral activation in both S1 and S2 regardless of which hand was stimulated, suggesting that these areas may be involved in integrating somatosensory input from both sides of the body. Some may attribute the involvement of ipsilateral S2 to a more cognitive role in sensory processing, and while it is important to consider the cognitive aspects of sensorimotor control, such as planning and strategy (106), bilateral S2 activation has been demonstrated in somatosensory studies regardless of the level of cognitive demand (18).

The involvement of S2 is particularly interesting in the context of aging, somatosensory dysfunction, and sensory rehabilitation. Age-related changes in activation have been seen, with decreased activation in S2 with tactile stimulation evident in elderly participants who are known to experience behavioral decline in somatosensory thresholds (54). The relationship of bilateral S2 with tactile sensation must also be considered in fields such as stroke research, where the location of the lesion has been demonstrated to impact both the type of somatosensory dysfunction (107), and also the ability to recover after stroke (108). Our finding of overlap in activation of right secondary somatosensory region for RH and LH tactile stimulation, may have particular relevance after stroke. For example, a stroke survivor with an infarct in the right hemisphere affecting S2 might not only experience the typically expected impairment of sensation in the contralateral hand (i.e., LH), but also impairment in the ipsilateral right hand; as has been described clinically (2). Further, recent evidence of altered functional connectivity in stroke survivors with impaired touch sensation following left or right hemisphere lesions, highlighted increased laterality indices in ipsilateral (contralesional) S2 relative to healthy controls following lesion of either hemisphere (109). Further, functional connectivity research has demonstrated that an increase in connectivity from contralesional S2 to contralesional thalamus correlates with better somatosensory function 6-months post-stroke (110).

Evaluation of the RH ROI studies (*n* = 12) revealed that only contralateral (left) S1 and bilateral S2 were examined by studies which predefined the areas thought to be involved in somatosensory processing of the hand. In comparison, the RH whole-brain studies also revealed anterior cingulate activation, and much larger clusters were involved with tactile stimulation. This suggests that when researchers set out to examine the functional activation of a tactile stimulus, if they limit the focus to a-priori areas, this may not capture the entire neural functional process related to the sensation. Anterior cingulate activation may play a significant role in sensory processing. For example, pleasant human touch is represented in anterior cingulate cortex (111). In addition, while attention differentially modulates signal amplitudes in the human somatosensory cortex, at higher intensities activation is also seen in the anterior cingulate cortex, consistent with attention to tactile stimuli in the current studies (112). It has been suggested that Von Economo (spindle) neurons found in cingulate cortex (113), and linked with insula, may have a role as part of a salience network (114). Network analyses identify anterior cingulate as a hub region and common co-activation of anterior cingulate and insula support the interpretation of a saliency network devoted to the integration of information from internal and external sensory environments (115). Further, interhemispheric connections between bilateral thalami occur via the anterior cingulate (113) and healthy controls show interhemispheric functional connectivity between a number of regions associated with somatosensory processing, including anterior cingulate (107), highlighting the contribution of both hemispheres and the broader somatosensory system. Interestingly, cingulate cortex has also been implicated in rats sensory recovery after lesions (116).

Other areas identified in this meta-analysis included inferior parietal lobe, insula, supramarginal gyrus and temporal lobe. Inferior parietal lobe (IPL) of the right hemisphere was identified for both RH whole-brain and ROI analyses. The location included OP1 and OP4. IPL has been associated with multi-modal sensory information integration (117, 118) and is reported to be part of the larger somatosensory network (119). The insula was also identified using the GingerALE peak maps, although this region was frequently assigned to the parietal operculum using the Anatomical toolbox. The insula has been identified as having a role in recognition, perception and learning in functional models of somatosensory processing (2). S2 is reciprocally connected with granular fields of the insula, reported to be devoted to somatic processing in monkeys (120). The close proximity of locations highlight the importance of the combined parietal opercular-insula region. Supramarginal gyrus is similarly located close to the parietal operculum/S2 region. The SMG is part of the somatosensory association cortex which has a role in interpretation of tactile sensory information as well as in perception of space and limbs location (121). Right SMG was found for RH whole-brain, RH ROI and LH whole-brain, and for the conjoined analyses. Right SMG is associated with spatial processing (121), consistent with tasks requiring localization of stimuli and/or involving spatial features of textures. Activation of left temporal gyrus, including auditory cortex and granular insula area 2, was greater in RH than LH whole-brain studies. Left temporal cortex has been linked with structural and semantic knowledge of body representation (122).

Each of the regions identified above have been implicated in stroke tactile impairment and recovery, potentially highlighting their broader importance. For example, change in functional connectivity from ipsilesional right S1 to right inferior parietal lobe was found in stroke survivors with impaired touch sensation compared to healthy controls (109). In addition, increased interhemispheric connectivity between the S2 region of interest and somatosensory association cortex (involving insula, parietal operculum and SMG) and temporal gyrus was found in healthy age-matched controls compared to stroke survivors with tactile deficits (109). Further, following tactile training, patients with lesions of sensory thalamus and/or internal capsule demonstrated activation in ipsiliesional insula, extending to the temporal pole, and supramarginal gyrus post-intervention (108). Interestingly, the regions identified have a role in the broader interpretation of tactile stimuli, including multi-modal integration, perception and learning, spatial processing and semantic knowledge and appear to be accessed as part of a wider somatosensory network.

There are limitations to this meta-analysis when examining the demographic information regarding the participants (see Table 1). Most of the LH studies (with the exception of one) included young participants (18–43 years). Aside from this, the cohorts were fairly well controlled, with the majority being RH dominant, and with tasks controlled for motor and other influences. Variable naming across studies can also contribute to confusion with interpretation. For example, terms such as secondary somatosensory cortex, secondary somatosensory region and secondary somatosensory area are often used interchangeably, although differences have been defined (10). To maximize accuracy and comparison across studies and broader literature in the field, we have reported on the MNI co-ordinates and peak location ALE results as well as the Anatomy Toolbox regional activation results.

The aim of this meta-analysis was to determine the convergence of foci reported from functional neuroimaging studies of touch sensation, separate to motor contributions and/or confounds. The findings advance our understanding of the separate, but potentially complementary, contributions of brain regions involved in processing touch sensation. Given the role of somatosensation and the somatosensory system in goal-directed actions of the upper limb and recovery after stroke, in depth knowledge of the role of key regions in the network is critical. The importance of bilateral S2 activation with right hand touch stimulation is highlighted, with a potential lateralization of activation in right S2 for right and left hand stimulation. This has implication for possible differences in unilateral vs. bilateral patterns of somatosensory impairment following right or left hemisphere lesion stroke. It may also identify a region with scope to contribute to recovery.

In conclusion, while research has established a role for S1 and S2 contralateral to the hand being stimulated (13, 14), this meta-analysis has demonstrated the need to also examine the bilateral activation in S2 with right hand stimulation in order to further delineate the role of this area in tactile processing. Additional studies examining LH tactile processing separate to the RH would be beneficial to further examine whether this same pattern of activation is seen. These two advances in understanding would in turn further research into somatosensory dysfunction and rehabilitation.

## Author Contributions

LC conceived the study. GL, LC, EL, and AB contributed to the design of the study. GL, SP, EL, and AB conducted the search and extraction of data. GL and PG conducted the meta-analysis. GL and LC interpreted the findings and drafted the manuscript. All authors critically reviewed and revised the manuscript for important intellectual content, provided approval for publication and agree to be accountable for all aspects of the work.

### Conflict of Interest Statement

The authors declare that the research was conducted in the absence of any commercial or financial relationships that could be construed as a potential conflict of interest.
